# What is an Allergist? *Reconciled Document Incorporating Member Society Comments, September 3, 2007*

**DOI:** 10.1097/wox.0b013e3181651679

**Published:** 2008-01-15

**Authors:** A position statement of the WAO Specialty and Training Council, Sergio Del Giacco, Lanny J Rosenwasser, Carlos D Crisci, Anthony J Frew, Michael A Kaliner, Bee Wah Lee, Liu Guanghui, Jorge Maspero, Hee-Bom Moon, Nokagawa Takemasa, Paul C Potter, Anand B Singh, Erkka Valovirta, Daniel Vervloet, John O Warner, Staff Liaison: Karen Henley

## 

An allergist is a physician who has successfully completed both a specialized training period in allergy and immunology and a training period in either internal medicine, or a sub-specialty of internal medicine such as dermatology, pneumology, or otorhinolaryngology, and/or pediatrics. Subject to national training requirements, allergists are also partially or fully trained as clinical immunologists, because of the immune basis of the diseases that they diagnose and treat. In most countries, the approved period of specialty training in allergy and immunology will be two to three years of specific, intense training. Depending on national accreditation systems, completion of this training will be recognized by a Certificate of Specialized Training in Allergy, in Allergy and Immunology, or in Allergy and Clinical Immunology, awarded by a governing board. In some countries this will follow successful completion of a certification test, and in other countries by competencies being signed-off by a training supervisor.

Fully trained allergists make an important contribution to designing local care systems and delivering the necessary care for patients with allergic diseases. Allergists act as advocates for patients, and support and argue the case for better education of the primary and secondary care physicians and other health care professionals who also care for allergic patients. Allergists should be available to provide care for the more complicated problems that are beyond the purview of well-trained primary and secondary care physicians and other health care professionals. The main defining characteristics of an allergist are the appreciation of the importance of external triggers in causing disease, and the knowledge of how to identify and manage these diseases, together with ex-pertise in appropriate drug and immunological therapies. This approach to diagnosis and therapy is a core value of the allergy specialist, and contrasts the allergist with many of the organ-based specialists whose patient bases may overlap with the specialty.

The unique training requirements for an allergist are detailed in "Requirements for Physician Training in Allergy: Key Clinical Competencies Appropriate for the Care of Patients with Allergic or Immunologic Diseases: A Provisional Position Statement of the World Allergy Organization."[1] In that document, the levels of allergy training required of first-, second-, and third-level physicians are documented, differentiating the training and knowledge base of an allergy specialist from that of primary care physicians and organbased specialists. The American Academy of Allergy, Asthma and Immunology has also published guidelines: "Consultation and Referral Guidelines Citing the Evidence: How the Allergist/Immunologist Can Help."[2] The European Union of Medical Specialists Allergy Training Syllabus [3,4] is available online at the World Allergy Organization Web site:http://www.worldallergy.org/allergy_certification/index.shtml.

## Defining the allergist

The allergist is a physician who has a broad range of highly specialized clinical and diagnostic skills and a strong knowledge base covering:

Disease manifestations, including those for every allergic, immunologic and overlapping disease, and the cellular basis of immune and allergic reactions;

Rhino-conjunctivitis

Sinusitis

Otitis

Asthma

Cough

Bronchitis

Hypersensitivity pneumonitis

Alveolitis

Atopic dermatitis/eczema

Contact dermatitis

Urticaria and angioedema

Drug allergy

Food allergy

Latex Allergy

Insect Allergy and Stinging-insect hypersensitivity

Gastrointestinal reactions resulting from allergy

Anaphylactic shock

Immunodeficiencies

Occupational allergic diseases

Risk factors for progression of allergic diseases--the "allergic march"

Other specific organ reactions resulting from allergy

Conditions that may mimic or overlap with allergic disease;

The epidemiology and genetics of allergic diseases and auto-immune diseases, and a specialist knowledge of regional and local allergens, including aero allergens, drugs, venoms, occupational allergens, and food allergens;

The effector cells involved in allergic disease (stem cells, lymphocytes, mast cells, basophils, eosinophils, neutrophils, monocytes, macrophages, dendritic cells);

The molecules involved in the immunological response (both innate and acquired), including chemical mediators; immunoglobulins; antibodies; complement; cytokines, including interleukins; chemokines and their receptors; and human leukocyte antigen/major histocompatibility complex (HLA/MHC) antigens;

The main hypersensitivity reactions;

Cell-to-cell interactions;

The scientific *in vitro *laboratory diagnostic tests for allergy andtheir selection and interpretation, including radio-allergosorbent tests (CAP-RAST^®^); enzyme_linked immunosorbent assays (ELISAs); Western blotting; tests for inflammatory markers (eosinophil cationic protein [ECP] and tryptase); cellular antigen stimulation tests (CAST^®^), and histamine release assays.

The allergist is especially competent in performing/interpreting the following:

Allergic history and physical examination

Skin testing

Ordering and interpreting allergy- and immunology-related laboratory tests

Evaluation of total IgE and allergen specific IgE measurements

Provocation testing for allergic and immunologic disease

Analysis and advice regarding local environmental/airborne allergens and irritants

Analysis and advice regarding ingested allergens/irritants

Conducting and/or evaluating tests of pulmonary function and tests of inflammatory markers

Conducting and/or evaluating tests of nasal function; this may include examination of nose and throat via fiberoptic rhinoscopy and nasal endoscopy

Specific allergen and venom immunotherapy

Pharmacotherapy of allergic disorders and related diseases

Where necessary, investigating alternative diagnoses

Environmental modification strategies to reduce allergen exposure

Immunomodulatory therapy

Drug desensitization

Evaluation and treatment of allergic and immunologic competence

Primary, secondary and tertiary prevention of allergic disease

Education for patients, caregivers and primary care physicians

## Appendix

All societies that have commented and approved

American Academy of Allergy, Asthma and Immunology

American College of Allergy, Asthma and Immunology

Argentine Association of Allergy and Immunology

Australasian Society of Clinical Immunology and Allergy

Bangladesh Society of Allergy and Immunology

Brazilian Society of Allergy and Immunopathology

Chilean Society of Allergy and Immunology

Colombian Allergy, Asthma, and Immunology Association

Danish Society for Allergology

Egyptian Society of Allergy and Clinical Immunology

French Society of Allergology and Clinical Immunology

German Society for Allergology and Clinical Immunology

Hungarian Society of Allergology and Clinical Immunology

Italian Society for Allergology and Clinical Immunology

Japanese Society of Allergology

Malaysian Society of Allergy and Immunology

Mexican College of Allergy, Asthma and Clinical Immunology

Mongolian Society of Allergology

Netherlands Society of Allergology

Paraguayan Society of Immunology and Allergy

Romanian Society of Allergology and Clinical Immunology

Russian Association of Allergology and Clinical Immunology

Allergy Society of South Africa

Singapore Society of Immunology, Allergy & Rheumatology

Swiss Society of Allergology and Immunology

Allergy and Immunology Society of Thailand

Turkish National Society of Allergy and Clinical Immunology

Venezuelan Society of Allergy and Immunology

Vietnam Association of Allergy, Asthma and Clinical Immunology

Zimbabwe Allergy Society

The Asia Pacific Association of Allergology and Clinical Immunology

Czech Society of Allergology and Clinical Immunology
